# Study to evaluate the safety and tolerability of *Streptococcus salivarius* eK12, a genetically modified strain derived from the oral probiotic S. *salivarius* K12: Results from a randomized, double-blind, placebo-controlled, parallel-group clinical trial

**DOI:** 10.3389/fnut.2025.1701611

**Published:** 2025-12-08

**Authors:** Francesco Di Pierro, Ali Akbar Bugti, Aasiya Bano, Mehtap Kara, Ikram Ujjan, Nasreen Mumtaz, Yasmeen Gull, Massimiliano Cazzaniga, Alexander Bertuccioli, Maria Laura Tanda, Nicola Zerbinati, Sumbal Hameed, Amjad Khan

**Affiliations:** 1 Velleja Research, Scientific Department, Milano, Italy; 2 Department of Medicine and Technological Innovation, University of Insubria, Varese, Italy; 3 Microbiota International Clinical Society, Turin, Italy; 4 Centre for Regenerative Medicine, University of Edinburgh, Edinburgh, United Kingdom; 5 Research & Innovation Unit, National Emergency Operation Centre (NEOC), Islamabad, Pakistan; 6 Department of Pharmaceutical Toxicology, Faculty of Pharmacy, Istanbul University, Istanbul, Türkiye; 7 Department of Pathology, Liaquat University of Medical and Health Sciences (LUMHS), Jamshoro, Pakistan; 8 Jam Ghulam Civil Hospital, Hub, Pakistan; 9 District Head Quarter Hospital, Turbat, Pakistan; 10 Department of Biomolecular Sciences, University of Urbino Carlo Bo, Urbino, Italy; 11 Endocrine Unit, Department of Medicine and Surgery, University of Insubria, Varese, Italy; 12 Department of Biochemistry, LUMHS, Jamshoro, Pakistan

**Keywords:** *Streptococcus salivarius* eK12, bacteriocins, engineered probiotics, genetically modified microorganisms, human safety, human tolerance

## Abstract

In recent years, genetically engineered probiotics have gained increasing scientific interest owing to their potential applications in human health and nutrition. *Streptococcus salivarius* K12 is a well-established oral probiotic with an excellent safety record, and a genetically modified derivative, *S. salivarius* eK12, has been recently developed to enhance its functional properties. The present study evaluated the safety and tolerability of *S. salivarius* eK12 administered as a dietary supplement in healthy adults. In a randomized, double-blind, placebo-controlled, parallel-group study conducted at the Jam Ghulam Qadir Civil Hospital, Hub, Pakistan (March–August 2025), 29 healthy adults (18–60 years) were assigned to receive *S. salivarius* eK12 (*n* = 13) or placebo (*n* = 16). Participants consumed one orally dissolving tablet twice daily (10 billion CFU/day) for 1 month, followed by 3 months of observation. This dose corresponded to approximately 10 times the level typically used in commercial probiotic formulations. Safety assessments included systemic, oral, and gastrointestinal examinations; self-reported questionnaires; vital signs; body weight; and laboratory investigations encompassing hematology, liver and renal function, electrolytes, HbA1c, thyroid profile, urinalysis, stool occult blood, bacterial culture, and inflammatory markers C-reactive protein (CRP), ferritin, and procalcitonin. All participants completed the study. *Streptococcus salivarius* eK12 was well-tolerated, with no serious adverse events (AEs) or discontinuations. Clinical, oral, and gastrointestinal examinations were unremarkable, and self-reported symptoms were minimal and comparable to placebo. Laboratory parameters and inflammatory markers remained within normal limits without clinically meaningful deviations. Stool and urine analyses were normal, and bacterial cultures were consistently negative. Extended follow-up revealed no delayed or cumulative adverse effects. These results provide the first clinical evidence that *S. salivarius* eK12, administered at 10 billion CFU/day for 1 month, is safe and well-tolerated in healthy adults. No adverse clinical or laboratory findings, and no evidence of infections or related complications, were observed during 4 months of monitoring, supporting its safe nutritional use and further clinical development.

**Clinical trial registration:**

https://clinicaltrials.gov/, identifier NCT06380270.

## 1 Introduction

Genetically engineered probiotics have recently garnered increasing scientific interest because of their potential applications in human health and nutrition (1–3). Advances in microbial genetics and synthetic biology have enabled the development of engineered or self-cloned probiotic strains with enhanced functional properties, such as improved antimicrobial activity, detoxification capacity, metabolic regulation, and immunomodulation. Examples include the autocloned *Lactobacillus reuteri* DSM 17938,which was derived from the parental ATCC 55730 strain by removing plasmid-carrying transferable antibiotic-resistance genes, thereby improving its biosafety profile (4); the engineered *Bacillus subtilis* ZB183, which expresses acetaldehyde dehydrogenase to metabolize toxic acetaldehyde and support hepatic detoxification (5, 6); the *Clostridium butyricum* pMTL007-GLP-1, which secretes glucagon-like peptide-1 for glycemic control and anti-diabetic effects (7); and probiotic vaccine prototypes based on *Lactobacillus* and *Streptococcus* vectors, which express conserved influenza A virus antigens to induce mucosal immune protection (8). Broader perspectives on these developments have been summarized in recent reviews (1, 3). Although commercialization of genetically engineered probiotics remains limited, such innovations represent an emerging frontier in the field of microbial therapeutics and nutritional science.

*Streptococcus salivarius* is a commensal species that naturally colonizes the human oral cavity from the first hours of life, contributing to microbial homeostasis and upper respiratory tract health (9, 10). Probiotics, including *S. salivarius*, play an important role in maintaining host health through multiple mechanisms, such as modulation of the microbiota and immune system, as highlighted in recent systematic reviews (11). In 1989, *S. salivarius* K12 (LMG P-27407), an oral probiotic that releases bacteriocins and induces interferon-*γ* production in the host oral cavity, was isolated from the tongue of a child who exhibited natural protection against the pathogenic bacteria *Streptococcus* *pyogenes* (12, 13). Its nutritional supplement use has demonstrated benefits in the prevention of recurrent *Streptococcus* pharyngitis, acute otitis media, periodic fever, aphthous stomatitis, pharyngitis, cervical adenitis syndrome (PFAPA), and viral upper respiratory tract infections, including SARS-CoV-2, in both children and adults (14–22). *Streptococcus salivarius* K12 is registered as a food supplement in the majority of countries in Europe, Australia, Asia, and the Americas and holds generally recognized as safe (GRAS) status in the United States, reflecting its safety history in both children and adults (10, 23, 24).

Scientific evidence indicates that *S. salivarius* K12 inhibits the growth of pathogenic bacteria in the oral cavity, including not only *S. pyogenes* but also *S. pneumoniae* and other pharyngeal pathogens, through competitive exclusion and the production of antimicrobial substances such as salivaricin A2 and salivaricin B (25–27). The ability of *S. salivarius* K12, particularly evident against oral pathogenic *streptococci*, is of particular interest for oral health due to the protection it offers against *streptococcal* infections, which are known to be recurrent, especially in pediatric settings. *S. salivarius* K12 forms a protective barrier against pathogens by colonizing the oral mucosa, thereby preventing their adhesion and subsequent colonization. Consequently, supplementation with *S. salivarius* K12 may reduce the frequency and severity of *streptococcal* infection episodes in children, potentially offering a safe and effective alternative to antibiotic therapy, while minimizing the risk of antibiotic resistance and microbiota disruption (11, 28, 29).

Despite these benefits, recent co-colonization studies using equal inocula of *S. salivarius* K12 and *S. pyogenes* have yielded unexpected findings (2). Under these conditions, activation of the quorum-sensing protein NIP in K12 was exploited by *S. pyogenes*, which responded by releasing the cysteine protease SpeB. This protease inactivated salivaricins A2 and B, thereby neutralizing the expected antagonistic effect of K12 To address this, a genetically modified derivative of the wild-type strain, termed *S. salivarius*, eK12, has been recently developed (2). This engineered strain was constructed by inserting a stop codon (ATG → TAG) in the quorum-sensing gene *nip* and replacing the native *sar* promoter with the constitutive native 235-bp *PtufA* promoter derived from the *tufA* gene of *S. salivarius* K12 (2). These modifications enhanced the expression of the *sar* biosynthetic gene cluster (sarBGC), increasing the production of salivabactin—a novel polyketide antibiotic—while preventing pathogen cross-signaling by *S. pyogenes*.

Although *S. salivarius* eK12 has exhibited enhanced antagonistic activity against *S. pyogenes* in experimental models, its safety and tolerability in humans have not yet been determined (2). As this strain is a genetically modified derivative of a commensal probiotic, rigorous safety evaluation is imperative before its broader use as a nutritional supplement for oral health. Such assessment is particularly important to rule out unforeseen adverse effects related to the introduced genetic modifications, including potential alterations in host–microbiota interactions or unexpected immune responses. To the best of our knowledge, this trial is the first-in-human clinical evaluation of a genetically modified *S. salivarius* strain. While the parental strain *S. salivarius* K12 has a well-established record of safety and efficacy, the safety of its engineered derivative *S. salivarius* eK12 has not previously been clinically characterized. This study therefore provides novel evidence demonstrating that *S. salivarius* eK12, administered at a dose that is approximately tenfold higher than that typically used for probiotic dietary supplements, is safe and well-tolerated in healthy adults—supporting its potential application in clinical nutrition and the translational development of genetically modified probiotics.

## 2 Materials and methods

### 2.1 Study design and setting

This study was a randomized, double-blind, placebo-controlled, parallel-group exploratory clinical trial conducted to evaluate the safety and tolerability of *S. salivarius* eK12 in healthy adults. Participants were enrolled at the Jam Ghulam Qadir Civil Hospital, Hub, Pakistan. Enrollment and follow-up visits were conducted between March and August 2025.

The study protocol was reviewed and approved by the Research Ethics Committee of Liaquat University of Medical and Health Sciences (Jamshoro, Pakistan; Ref. No. LUMHS/REC/-497/11.11.2024) and the Ethical Review Committee of Mekran Medical College (Turbat, Pakistan; Ref. No. MMC/REC/3.3.2025). The trial adhered to the principles of the Declaration of Helsinki and Good Clinical Practice (GCP). Written informed consent was obtained from all participants prior to enrollment. The trial was registered on https://www.ClinicalTrials.gov (identifier: NCT06380270).

### 2.2 Participants and eligibility criteria

Healthy adult volunteers of either sex, aged 18–60 years, were eligible for inclusion. Participants were required to have no underlying medical conditions, a body mass index (BMI) between 18.5 and 35 kg/m^2^, and no known allergies or intolerance to probiotics. Baseline hematology, clinical chemistry, urinalysis, and vital signs had to be within clinically acceptable ranges.

The exclusion criteria included the following:

1. Severe allergies to probiotics
2. Recent oral surgery or dental procedures
3. Active dental disease or systemic oral conditions
4. Immunodeficiency or immunosuppressive therapy
5. Pregnancy or breastfeeding
6. Smoking within the past 6 months
7. Recent antibiotic use (within 4 weeks)
8. Gastrointestinal disorders, unstable metabolic disease, heart failure, or any chronic systemic illness.

### 2.3 Randomization and blinding

Eligible participants were randomized in a 1:1 ratio to the probiotic eK12 or placebo group using a computer-generated sequence. Allocation was concealed, and participants, investigators, and study staff remained blinded to group assignment.

### 2.4 Intervention

Participants underwent 1 month of administration of one orally dissolving tablet, taken twice daily. The probiotic tablet contained *S. salivarius* eK12 (Bactoblis^®^ Evol, Pharmextracta S.p.A., Pontenure, Italy), a self-cloned (autocloned) derivative of the parental *S. salivarius* K12 strain previously developed and characterized by Do et al. (2). This strain was engineered to enhance antimicrobial activity and prevent pathogen signal interference through the insertion of a stop codon in the *nip* gene and the substitution of the quorum-regulated *sar* promoter with the constitutive native *pTufA* promoter, thereby increasing salivabactin production. No foreign or synthetic DNA sequences were introduced, and all genetic elements originate from the parental genome. The strain was confirmed to be genetically stable for over 100 generations with no plasmid mobility or fitness cost and poses minimal risk of environmental dissemination (2). In accordance with EU Directive 2009/41/EC (Annex II, Part A), such autocloned microorganisms are not classified as genetically modified microorganisms (GMMs) and are handled under biosafety level 1 (BSL-1) conditions. In 2024, *S. salivarius* eK12 was registered as a dietary supplement with the Italian Ministry of Health (notification no. I.5.i.h.2/2024/177553, dated 24 July 2024) under the commercial name Bactoblis^®^ Evol (Pharmextracta S.p.A., Pontenure, Italy), supporting its recognition for use as a food supplement within the European Union.

Each probiotic tablet contained approximately 5 × 10^9^ viable eK12 cells at the time of manufacture, corresponding at the expire date to a value not < to 1 × 10^10^ CFU (10 billion viable cells). Such a tablet was administered during the intervention period. A supra-physiological dose of *S. salivarius* eK12 (10 billion CFU/day), approximately tenfold higher than conventional probiotic supplementation levels, was selected to ensure robust safety evaluation in this first-in-human study. The placebo consisted of identically matched orally dissolving tablets that were identical in appearance, taste, and formulation excipients, but without the active probiotic strain. Participants were instructed to allow each tablet to dissolve slowly in the mouth with lips closed, without chewing or swallowing whole, and to avoid eating, drinking, or brushing their teeth immediately afterward.

### 2.5 Clinical assessments

Clinical evaluations were performed at baseline, after 1 month of administration, and at 2- and 4-month follow-up visits. Each visit included a general physical examination (skin, eyes, ears, nose, throat, cardiovascular, respiratory, abdominal, musculoskeletal, lymphatic, and neurological systems), body weight measurement, and vital signs assessment (blood pressure, heart rate, respiratory rate, and temperature).

Oral examinations included inspection of the pharynx, tongue, teeth, gums, and mucosa for inflammation, ulceration, or infection.

Participants also completed an Oral and Gastrointestinal Health Assessment Questionnaire at each visit using a 0–10 visual analog scale (VAS) approach to evaluate oral (tooth sensitivity, gum bleeding, and halitosis) and gastrointestinal (abdominal pain, bloating, heartburn, nausea, vomiting, diarrhea, and flatulence) symptoms. The questionnaire and the scoring system were adapted from the methodology described by Burton et al., which used a similar 0–10 VAS approach to assess oral and gastrointestinal tolerance in human safety studies of *S. salivarius* K12 (24). Each symptom was rated on a 0–10 scale, where 0 indicated no symptom (normal), 1–3 indicated mild occasional discomfort, 4–6 indicated moderate noticeable discomfort, and 7–10 indicated severe, constant symptoms significantly impacting daily life.

Adverse events (AEs) were recorded at every study visit and through participant self-reports. AEs were defined and categorized in accordance with the International Council for Harmonisation (ICH) E6(R2) and Medical Dictionary for Regulatory Activities (MedDRA) guidelines as any untoward medical occurrence, regardless of the causal relationship. Serious adverse events (SAEs) were defined as events resulting in death, hospitalization, life-threatening conditions, or persistent disability. All AEs were assessed by the study physician for intensity (mild, moderate, and severe), causality, and outcome, and they were followed until they resolved or stabilized.

### 2.6 Laboratory investigations

Laboratory tests were performed at baseline and at the 4-month follow-up visits. These included the following:

1. *Hematology:* Complete blood count (CBC)
2. *Biochemistry:* Liver function tests (alanine aminotransferase (ALT), aspartate aminotransferase (AST), alkaline phosphatase (ALP), and bilirubin), renal function tests (creatinine and blood urea), and serum electrolytes
3. *Metabolic profile:* HbA1c and thyroid function (T3, T4, TSH)
4. *Urinalysis and stool occult blood tests:* To assess renal and gastrointestinal health
5. *Inflammatory markers:* C-reactive protein (CRP), ferritin, and procalcitonin (PCT) to monitor for any systemic inflammatory or immune-mediated responses.
6. *Microbiological evaluation:* Blood cultures tests were performed to assess potential systemic bacterial infection and confirm the absence of treatment-related bacteremia.

### 2.7 Outcome measures

The primary outcome was the safety and tolerability of *S. salivarius* eK12 administration in healthy adults. Safety was assessed through adverse event monitoring, clinical and oral examinations, gastrointestinal assessments, laboratory parameters, and vital signs. An extended follow-up at 2 and 4 months was conducted to detect any delayed or cumulative effects.

### 2.8 Statistical analysis and sample size

All analyses were conducted using descriptive and exploratory inferential statistics. Continuous variables were summarized as mean ± standard deviation (SD), and categorical variables were presented as counts and percentages. The distribution of continuous variables was assessed using the Shapiro–Wilk test to evaluate normality. Between-group and within-group comparisons were performed across study visits using repeated-measures analysis of variance (ANOVA) for normally distributed data or the non-parametric Friedman and Mann–Whitney *U* tests when normality assumptions were not met. For categorical variables, differences between groups were assessed using the chi-squared test or Fisher’s exact test, as appropriate. Given the exploratory and safety-oriented nature of this first-in-human clinical study, no correction for multiple comparisons was applied, and *p*-values were interpreted descriptively. A two-tailed *p*-value of < 0.05 was considered nominally significant. Statistical analyses were performed using SPSS Statistics version 27.0 (IBM Corp., Armonk, NY, USA) and GraphPad Prism version 9.0 (GraphPad Software, San Diego, CA, USA).

No formal sample size calculation was performed because the primary objective of this study was to characterize the safety and tolerability profile of *S. salivarius* eK12. The inclusion of 29 participants (13 receiving *S. salivarius* eK12 and 16 receiving placebo) was considered adequate to detect any clinically relevant safety or tolerability signals in this exploratory setting. The assumed safety of the eK12 strain was supported by preclinical toxicological data (unpublished)—including the Ames test (OECD 471), acute oral toxicity (OECD 423), and 90-day repeated-dose oral toxicity (OECD 408) studies in rats—which revealed no adverse findings. This assumption was further reinforced by the established human safety profile of the parental *S. salivarius* K12 strain, as demonstrated by previously reported studies (24, 30). Accordingly, the statistical approach focused on clinical, rather than purely statistical, significance, emphasizing the absence of adverse effects and the overall tolerability profile of *S. salivarius* eK12 rather than the detection of group differences or efficacy-related outcomes.

## 3 Results

**Figure 1:**
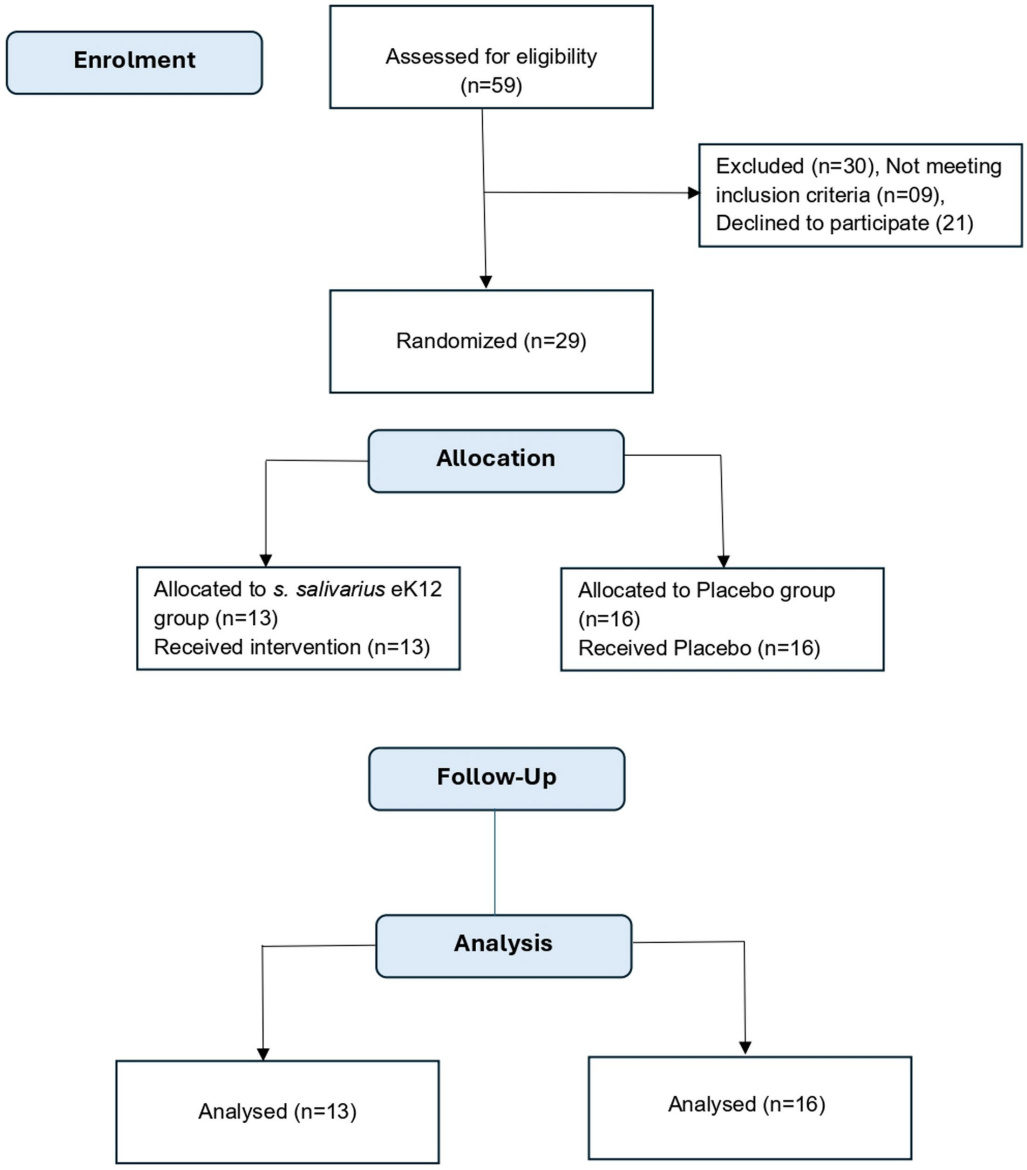
Study CONSORT flow diagram.

A total of 29 participants were randomized, with 13 allocated to the *S. salivarius* eK12 group and 16 to the placebo group (Figure 1). Baseline demographic and socioeconomic characteristics were comparable between the two groups (Table 1). The mean age was 30.9 ± 9.9 years in the eK12 group and 29.9 ± 10.7 years in the placebo group. Sex distribution was balanced, with a nearly equal proportion of men and women in both groups. The mean BMI was 23.6 ± 3.1 kg/m^2^ in the eK12 group and 24.3 ± 3.5 kg/m^2^ in the placebo group. The majority of participants had completed secondary or higher education and were from lower- to middle-socioeconomic backgrounds. Marital status distribution was also similar across the two groups. None of the participants reported any history of underlying medical conditions, concurrent medication use, alcohol consumption, tobacco use, or contraceptive use.

**Table 1:** Baseline demographic and socioeconomic characteristics of participants.

Characteristic	*eK12* group (*n* = 13)	Placebo group (*n* = 16)
Age, years (mean ± SD, range)	30.9 ± 9.9 (18–45)	29.9 ± 10.7 (18–48)
Sex, *n* (%)	Male: 7 (53.8%) Female: 6 (46.2%)	Male: 8 (50.0%) Female: 8 (50.0%)
BMI, kg/m^2^ (mean ± SD, range)	23.6 ± 3.1 (18.5–29.4)	24.3 ± 3.5 (19.5–32.0)
Marital status	Married: 8 Unmarried: 5	Married: 10 Unmarried: 6
Education level	Majority secondary or higher	Majority secondary or higher
Socioeconomic status	Predominantly lower to middle income	Predominantly lower to middle income

### 3.1 General physical examination

During all follow-up visits, the majority of participants in both groups demonstrated normal findings on general physical examination. Skin assessments identified a few cases of mild atopic dermatitis in the placebo group at the 2- and 4-month visits, coinciding with a community outbreak and considered unrelated to study administration. Isolated cases of conjunctivitis were reported in both groups at the 1- and 4-month visits, also attributed to local outbreak patterns. One participant in the placebo group experienced transient ear pain at the 4-month visit, which resolved spontaneously. Occasional mild seasonal flu episodes with low-grade fever were observed in both groups at the 1- and 2-month visits; these were self-limiting and unrelated to the intervention. Cardiovascular and respiratory assessments, including heart sounds and lung auscultation, were normal throughout the study. Abdominal, musculoskeletal, lymph node, and nervous system examinations were consistently normal at each visit. Body weight and vital signs, such as blood pressure, heart rate, respiratory rate, and temperature, remained within clinically acceptable ranges, with no clinically meaningful changes from baseline to follow-up.

### 3.2 Oral examination

Oral examinations were unremarkable across all visits. Pharyngeal inspection and dental examinations were normal in both groups. One participant in the eK12 group reported oral thrush at the 4-month follow-up visit, consistent with a prior history of intermittent thrush; this was not considered study-related. Gingival health and oral mucosa assessments were consistently normal, with no clinically significant bleeding or pathology reported.

### 3.3 Oral and gastrointestinal health assessment

Oral health questionnaires, including teeth sensitivity, gum bleeding, and bad breath scores (VAS 0–10), remained within normal levels across all participants. Gastrointestinal health scores, covering abdominal pain, bloating, heartburn/reflux, nausea, vomiting, diarrhea, and flatulence, were also consistently normal across visits. Occasional mild symptoms, such as transient reflux, mild bloating, or nausea, were reported sporadically in both groups but were self-limiting and judged to be unrelated to study administration.

### 3.4 Serious adverse events

No participant experienced any serious adverse events during the study.

### 3.5 Laboratory investigations

#### 3.5.1 Hematology

**Figure 2:**
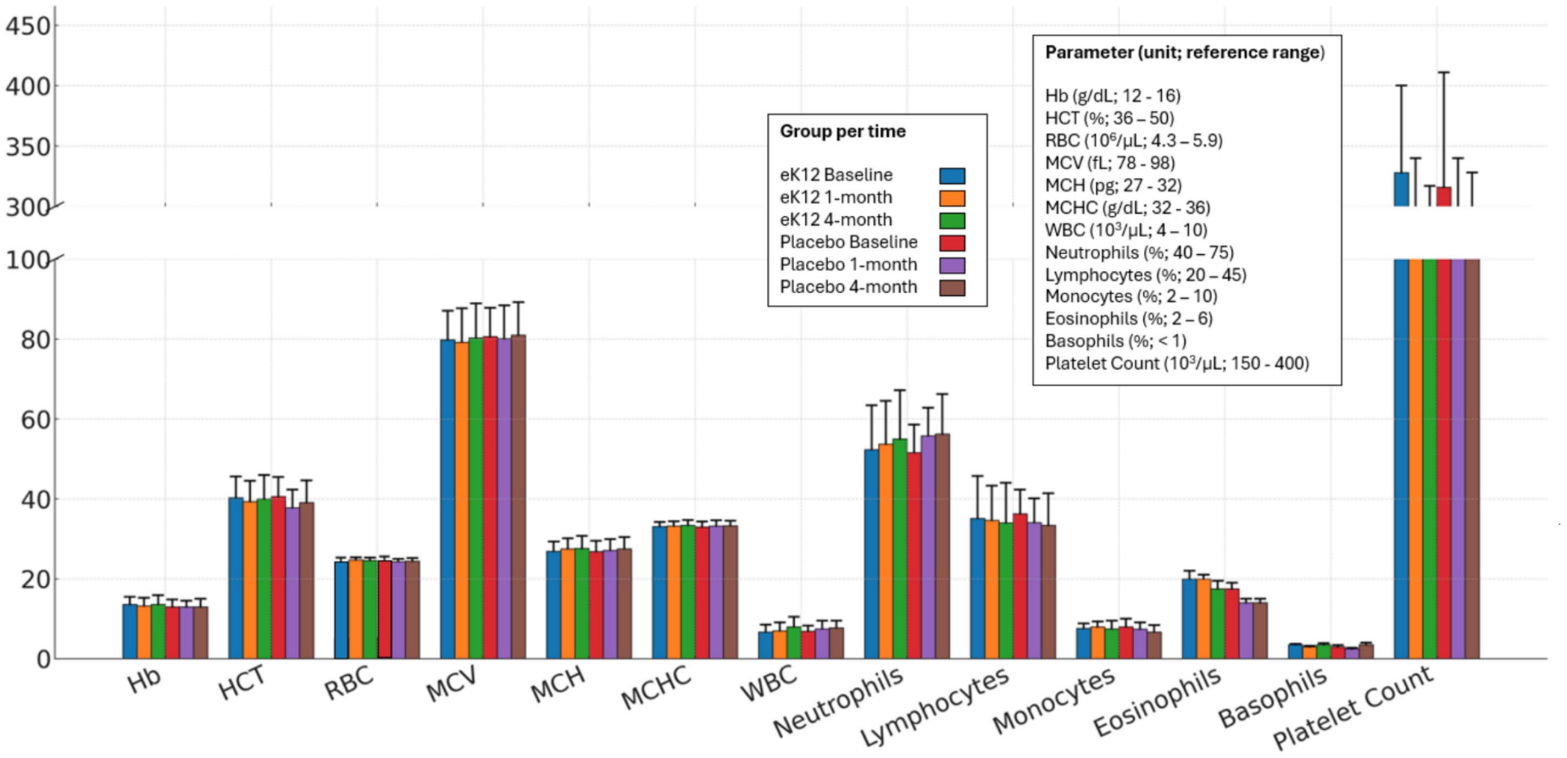
Comparison of complete blood count (CBC) parameters between the eK12 (*n* = 13) and placebo (*n* = 16) groups at baseline, 1-month, and 4-month follow-up visits. Values are presented as mean ± SD. Hb, hemoglobin; HCT, hematocrit; RBC, red blood cells; MCV, mean corpuscular volume; MCH, mean corpuscular hemoglobin; MCHC, mean corpuscular hemoglobin concentration; WBC, white blood cells.

Complete blood count (CBC) parameters remained within normal reference ranges across both groups at all follow-ups (Figure 2, Supplementary Table S1). No clinically significant changes were observed in hemoglobin, hematocrit, white cell count, differential counts, or platelet levels between baseline and follow-up visits.

#### 3.5.2 Liver function tests

**Figure 3:**
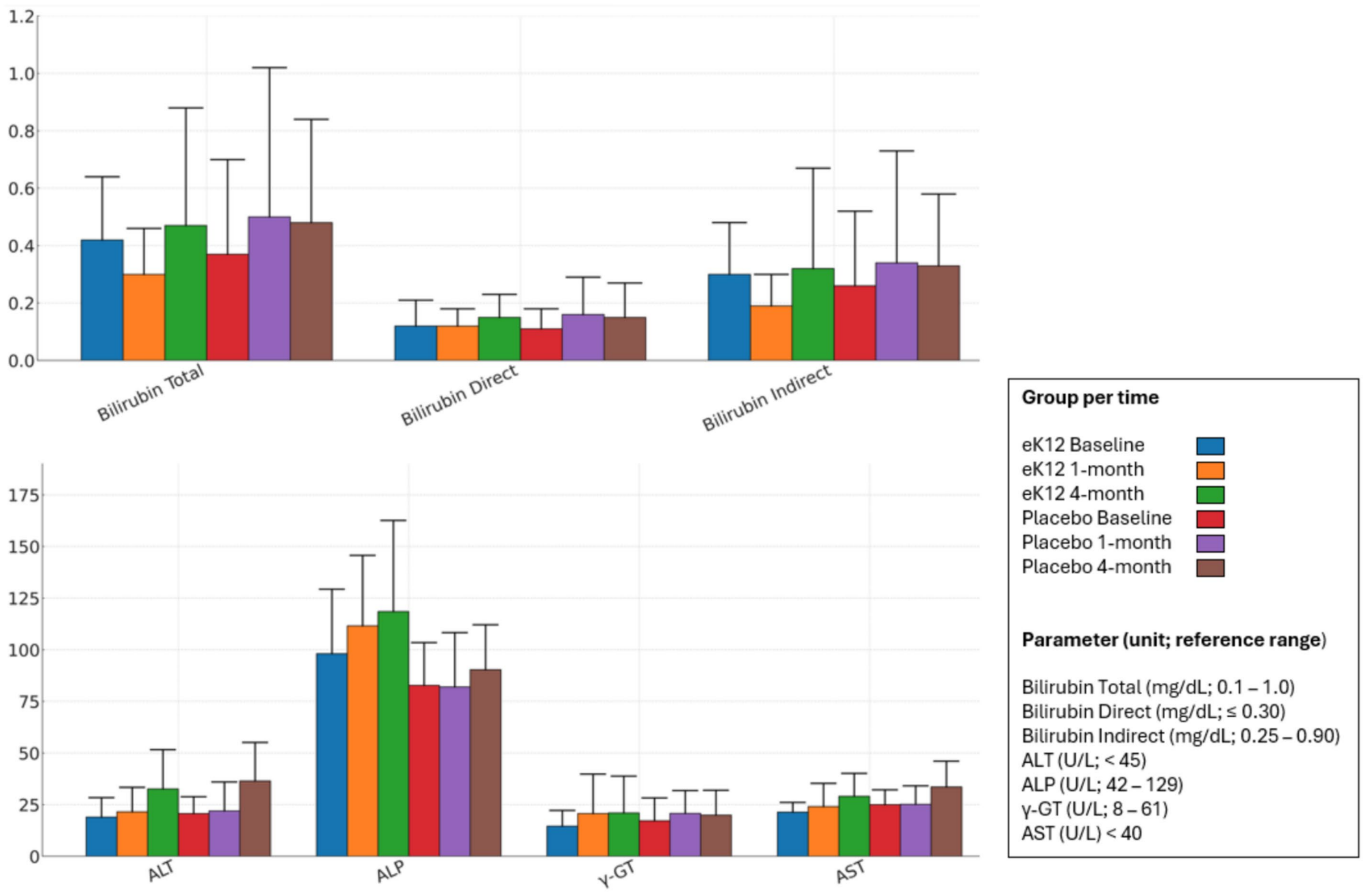
Liver function test parameters in the eK12 (*n* = 13) and placebo (*n* = 16) groups at baseline, 1-month, and 4-month follow-ups. Values are presented as mean ± SD. ALT, alanine aminotransferase; ALP, alkaline phosphatase; *γ*-GT, gamma-glutamyl transferase; AST, aspartate aminotransferase.

Serum liver function parameters, including bilirubin (total, direct, and indirect), ALT, AST, ALP, albumin, and total protein, remained within reference ranges across both groups at all time points (Figure 3, Supplementary Table S2). Minor fluctuations were observed in individual values over time; however, there were no consistent trends or clinically meaningful differences between the *S. salivariu* eK12 and placebo groups. No evidence of hepatotoxicity or liver-related adverse effects was detected during the intervention or follow-up period.

#### 3.5.3 Renal function tests

**Figure 4:**
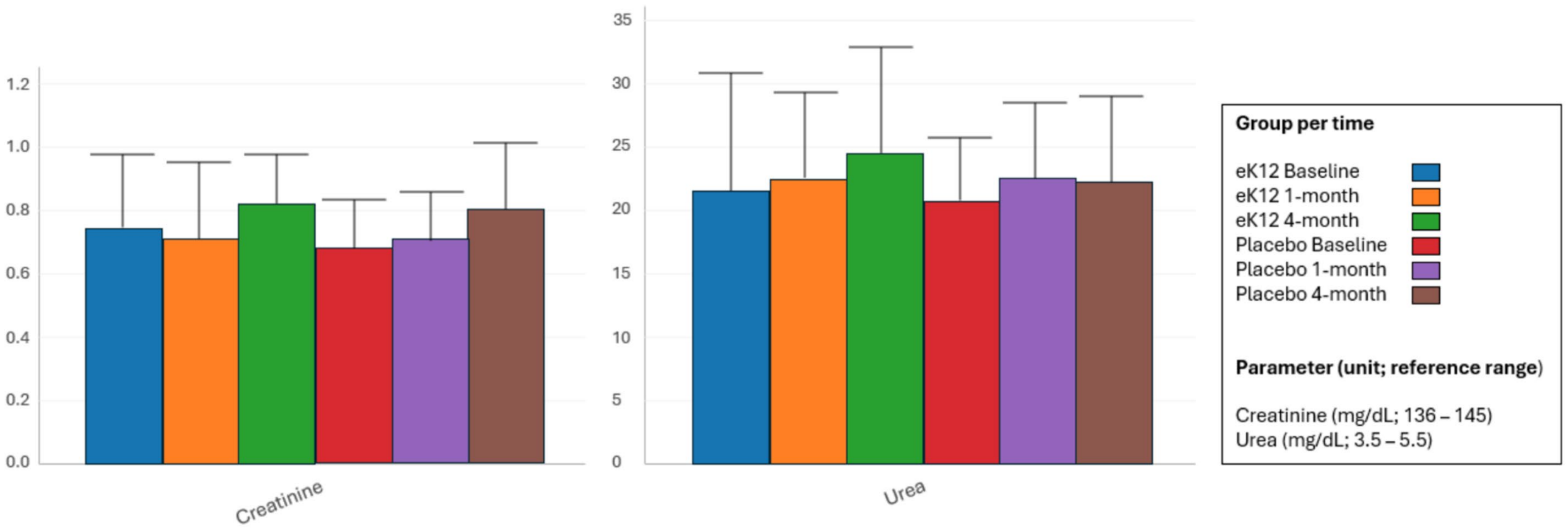
Renal function test parameters in the eK12 (*n* = 13) and placebo (*n* = 16) groups at baseline, 1-month, and 4-month follow-ups. Values are presented as mean ± SD.

Serum creatinine and blood urea levels remained within normal reference ranges across both the *S. salivarius* eK12 and placebo groups from baseline to the 4-month follow-up visits (Figure 4, Supplementary Table S3). Minor fluctuations were observed at individual time points, but these were not clinically significant and showed no consistent trend attributable to the study intervention. Overall, renal function parameters were stable throughout the study in both groups, with no evidence of treatment-related renal impairment.

#### 3.5.4 Electrolytes

**Figure 5:**
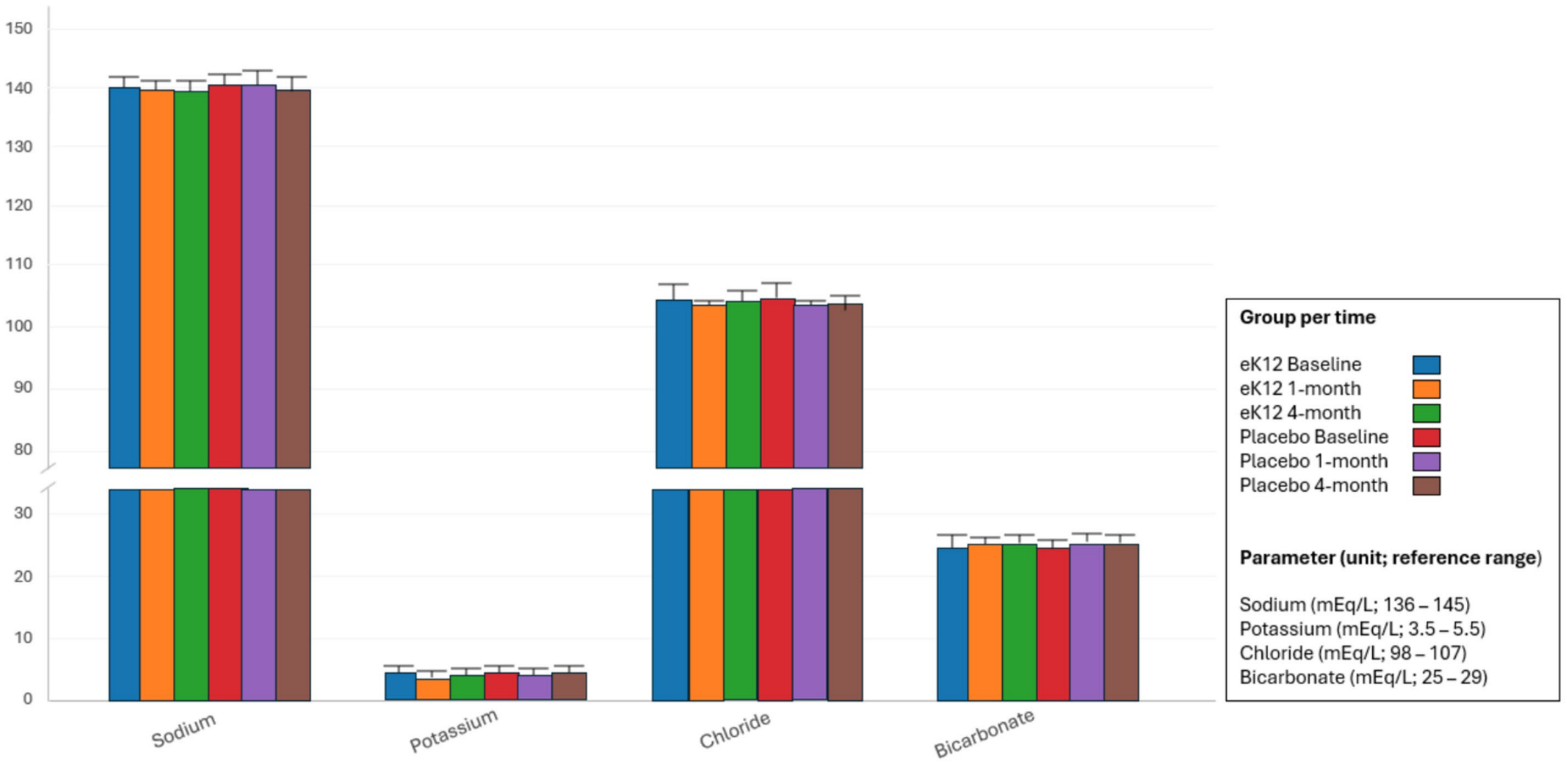
Comparison of serum electrolytes between the eK12 (*n* = 13) and placebo (*n* = 16) groups at baseline, 1-month, and 4-month follow-ups. Values are presented as mean ± SD.

Serum electrolyte levels, including sodium, potassium, chloride, and bicarbonate, remained within their respective reference ranges across all study visits in both the *S. salivarius* eK12 and placebo groups and showed no clinically significant differences between the two groups or over time (Figure 5, Supplementary Table S4). Occasional minor fluctuations were observed in individual participants (e.g., transient low-normal sodium or borderline high bicarbonate), but these were sporadic, not dose-dependent, and were clinically insignificant. Overall, electrolyte profiles remained stable, indicating no safety concerns associated with the administration *of* eK12.

#### 3.5.5 Blood glycemic level

**Figure 6:**
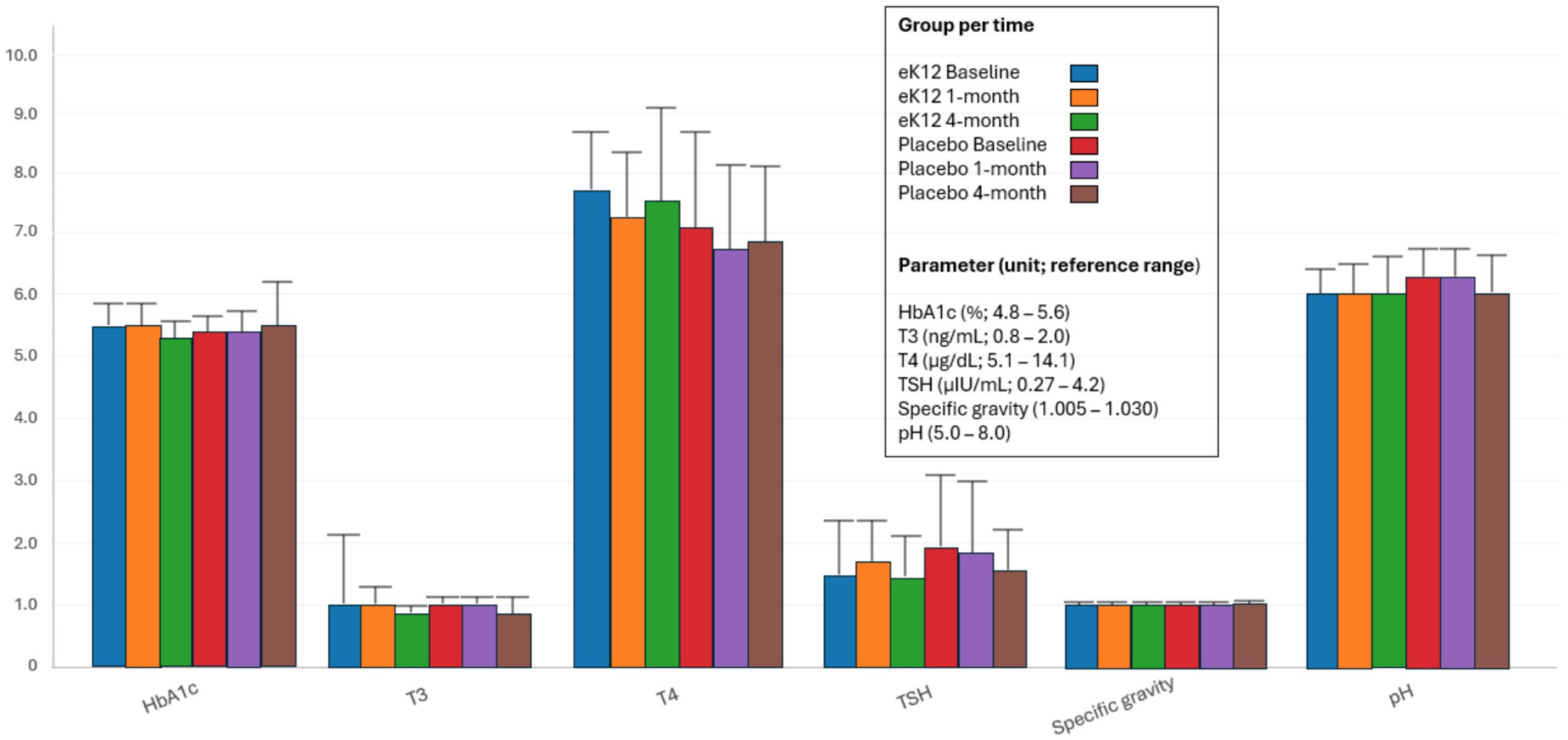
Comparison of HbA1c, hemoglobin A1c, function parameters, triiodothyronine (T3), thyroxine (T4), thyroid-stimulating hormone (TSH), and urinalysis between the eK12 (*n* = 13) and placebo (*n* = 16) groups at baseline, 1-month, and 4-month follow-up visits. Values are presented as mean ± SD.

HbA1c levels in both groups remained stable and within or near the normal reference range (4.8–5.6%) (Figure 6, Supplementary Table S5). No significant differences were observed between the eK12 and placebo groups throughout the 4-month follow-up, indicating no adverse effects of eK12 administration on glycemic control.

#### 3.5.6 Thyroid function

Mean T3, T4, and TSH levels remained within the normal reference ranges in both groups at all time points (Figure 6, supplementary Table S6). Overall, no clinically meaningful differences were observed between the two groups, indicating that *S. salivarius* eK12 supplementation had no adverse effects on thyroid function.

#### 3.5.7 Stool occult blood test

All participants in both the eK12 and placebo groups tested negative for stool occult blood at baseline and the 1-month visit. At the 4-month visit, one participant in the eK12 group and one in the placebo group tested positive. These isolated findings were not considered related to the study intervention.

#### 3.5.8 Urinalysis

Urine physical, chemical, and microscopic parameters were largely within normal limits across both the eK12 and placebo groups at all follow-up visits (Figure 6, Supplementary Table S7). Color and appearance were generally normal (pale yellow/yellow and clear), with only occasional dark yellow or slightly turbid samples, which were not clinically significant. Chemical examination showed that glucose, ketones, bilirubin, and urobilinogen were consistently negative or within normal limits, while trace or 1 + proteinuria appeared sporadically in both groups. Microscopic findings, such as red blood cells (RBCs), leukocytes, epithelial cells, and bacteria, were mostly within the reference range (0–2/HPF) and scattered without consistent trends. Yeast, casts, and crystals were rarely observed and appeared as incidental findings. Overall, no negative effects of *S. salivarius* eK12 supplementation on urine parameters were observed compared to the placebo group.

#### 3.5.9 Culture blood tests

Bacterial cultures were negative in all participants across both groups at all study visits, confirming the absence of bacteremia or systemic bacterial infection.

#### 3.5.10 Inflammatory markers

**Figure 7:**
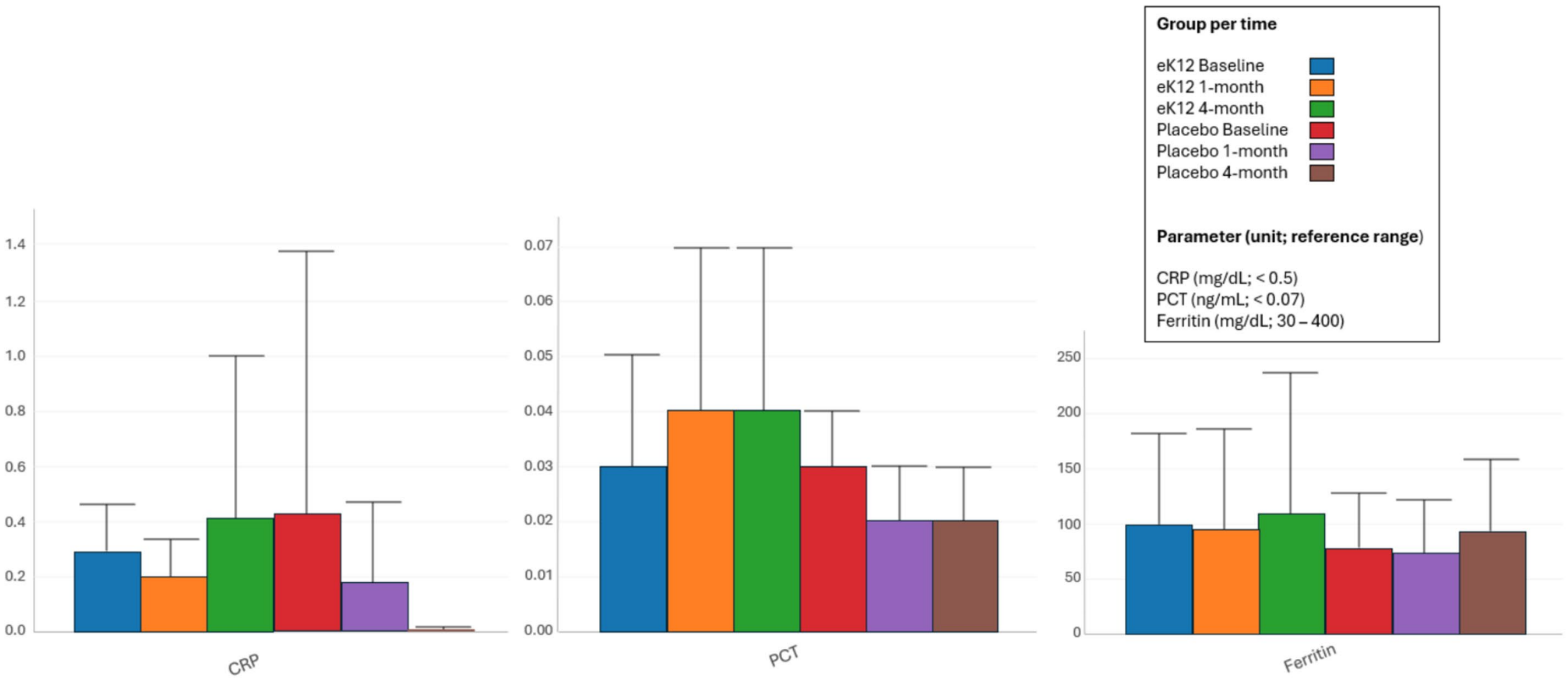
Serum inflammatory marker levels in the eK12 (*n* = 13) and placebo (*n* = 16) groups at baseline, 1-month, and 4-month follow-ups. Values are presented as means ± SD. C-reactive protein; PCT, procalcitonin.

Serum inflammatory markers remained within normal reference ranges throughout the 4-month follow-up in both groups (Figure 7, Supplementary Table S8). C-reactive protein (CRP) levels (<0.5 mg/dL) showed no consistent or clinically meaningful elevations, and procalcitonin (PCT) values (<0.07 ng/mL) were stable across all time points, indicating no evidence of bacterial infection or systemic inflammatory activation. Ferritin concentrations also remained within expected physiological limits without patterns suggestive of inflammation. Collectively, these findings confirm that *S. sali*var*ius* eK12 supplementation was not associated with systemic inflammatory responses.

A cluster heatmap summarizing mean values of hematological, biochemical, and urinary parameters at baseline, 1-month, and 4-month follow-ups for both the *S. salivarius* eK12 and placebo groups is presented in Supplementary Figure S1. Green indicates values within the normal reference ranges, whereas yellow denotes minor, non-pathological variations. No clinically relevant deviations were observed, confirming the overall stability and safety of *S. salivarius* eK12 administration.

## 4 Discussion

This randomized, double-blind, placebo-controlled exploratory trial evaluated the safety and tolerability of a genetically modified *S. salivarius* eK12 strain in healthy adults. To the best of our knowledge, this is the first human study of this genetically modified probiotic, and the findings indicate that daily supplementation with *S. salivarius* eK12 (1 × 10^10^ CFU/day for 1 month), a dose that is approximately tenfold higher than the labeled daily dose of commercial *S. salivarius* K12 oral formulations, was safe and well-tolerated, with no adverse clinical or laboratory effects observed during the 4-month monitoring period.

Clinical evaluations, including general physical, oral, and gastrointestinal examinations, as well as body weight and vital signs, showed no abnormalities or concerning trends. Questionnaire-based assessments confirmed that oral and gastrointestinal symptoms remained minimal and comparable to placebo, with no indication of intolerance. Laboratory tests, including hematology, biochemistry, liver and renal function, electrolytes, HbA1c, thyroid function, and urinalysis, remained within physiological limits. Importantly, inflammatory biomarkers—C-reactive protein, ferritin, and procalcitonin—showed no evidence of systemic inflammatory activation. Stool occult blood and bacterial cultures were consistently negative. Extended follow-up at 2 and 4 months confirmed that no delayed or cumulative adverse effects occurred.

These results are consistent with the established safety of the wild-type *S. salivarius* K12 strain (14–22, 24, 30), which has long been used as a probiotic dietary supplement for oral health protection against pathogenic GAS bacteria with an excellent safety record. Importantly, this study demonstrates that the genetic modification of *S. salivarius* K12 did not introduce additional safety concerns, an important finding in the context of genetically engineered probiotics. These findings, together with the extensive historical safety record of the parental K12 strain, reinforce the translational potential and safe nutritional application of the modified *S. salivarius* eK12 strain. From a regulatory and ethical perspective, the eK12 strain qualifies as an autocloned microorganism under EU Directive 2009/41/EC and is handled at Biosafety Level 1; its commercial registration as a dietary supplement in Italy further supports its recognized safety status within the European Union.

Supporting preclinical data also reinforce this conclusion. We have recently conducted complementary toxicology studies with *S. salivarius* eK12, including the *in vitro* Ames test (OECD 471) for genotoxicity, a GLP-compliant acute oral toxicity study (OECD 423), and a 90-day repeated-dose toxicity study (OECD 408) in Wistar rats (24). Although unpublished, these studies revealed no adverse findings, aligning with the human safety outcomes reported here.

From an ecological perspective, the engineered *S. salivarius* eK12 strain behaves identically to the parental K12 strain, maintaining comparable growth, colonization dynamics, and host–microbiota compatibility (2). Preclinical investigations reported that eK12 does not exhibit increased ecological fitness or altered microbiome interactions in oral, nasopharyngeal, or vaginal niches and remains genetically stable with no plasmid mobility or horizontal transfer potential (2). The modest upregulation of salivabactin production does not extend its inhibitory spectrum beyond pathogenic competitors such as *S. pyogenes* and *S. pneumoniae*. Furthermore, the currently available literature indicates that *S. salivarius* species are recognized as safe commensals that support microbial homeostasis in the oral and gastrointestinal ecosystems (9, 10, 31–33).

Given that recurrent pharyngitis due to *S. pyogenes* is most prevalent in children, the evaluation of *S. salivarius* eK12 in this population is of particular importance. *S. salivarius* is a predominant human commensal of the oropharynx and may constitute a large proportion of the oral bacterial population (32). It has also been abundantly detected in human breast milk and in the small intestine (34, 35). The persistent natural exposure of humans to large numbers of *S. salivarius* shortly after birth supports the hypothesis that re-exposure from external sources is potentially harmless through probiotic preparations containing strains such as K12 or M18 (30, 31). The K12 strain, in particular, has been extensively studied for oral health benefits, including reducing recurrences of *S. pyogenes* pharyngotonsillitis (11, 28). Recurrent bacterial throat infections in children are often managed with antibiotics, but repeated or prolonged use is associated with major drawbacks, including antimicrobial resistance, adverse drug reactions, and disruption of the microbiota (36–38). In some cases, antibiotics fail to completely eradicate *streptococci*, resulting in persistent carriage or recurrent infections. These limitations have generated increased interest in alternative preventive strategies, such as precision probiotics, including those targeting oral streptococcal infections with strain K12 (39). This finding aligns with recent evidence emphasizing the promise of natural and probiotic-based interventions as effective alternatives to conventional antibiotics in combating bacterial infections, including urinary and respiratory pathogens (40). Since children are disproportionately affected by recurrent pharyngotonsillitis and possess developing immune systems, future clinical research should emphasize the safety evaluation of eK12 in pediatric populations.

Strengths of this trial include its randomized, double-blind, placebo-controlled design; structured multi-system safety monitoring; administration of a supra-physiological dose; and extended follow-up.

A key limitation of this study is the modest sample size (*n* = 29), which restricts statistical power and the ability to detect rare adverse events. However, the extensive clinical safety record of the parental *S. salivarius* K12 strain, along with the fact that *S. salivarius* is a qualified presumption of safety (QPS)-listed species in Europe and is generally recognized as safe (GRAS) by the U.S. Food and Drug Administration (FDA), provides strong regulatory and historical support for its safe use in humans. In addition, unpublished GLP-compliant, OECD-guided preclinical studies for *S. salivarius* eK12—including mutagenicity (OECD Test No. 471), acute oral toxicity (OECD Test No. 423), and 90-day repeated-dose toxicity (OECD Test No. 408)—confirmed the absence of toxic or genotoxic effects. Based on these findings, the sample size was deemed appropriate to descriptively evaluate short-term tolerability under conditions of exaggerated exposure—approximately tenfold higher than the labeled daily dose of commercial *S. salivarius* K12 formulations. Another limitation of this study is the absence of microbiota profiling and immunological endpoints (e.g., cytokines and secretory IgA). Although these assessments were beyond the scope of this first-in-human safety trial, future studies should incorporate metagenomic and immunological analyses to better characterize host–microbe and immune interactions associated with *S. salivarius* eK12.

Another limitation of this study is that it was conducted exclusively in adults, whereas the probiotic is primarily intended for pediatric use. Although extensive clinical data support the safety of the parental *S. salivarius* K12 strain in children, age-related differences in immune maturation and microbiota composition could theoretically affect tolerance and colonization dynamics. Future studies should therefore include pediatric cohorts to confirm safety and tolerability in this target population.

Nevertheless, this study provides the first clinical safety data for a genetically modified *S. salivarius* strain administered at a dose that is approximately tenfold higher than that typically used in probiotic dietary supplements, with no adverse clinical, laboratory, or microbiological effects observed.

In conclusion, this study provides the first clinical evidence that *S. salivarius* eK12, administered as a dietary supplement at a high dose relative to the labeled daily intake of *S. salivarius* K12 in standard oral probiotic products, is safe and well-tolerated in humans. Together with supporting GLP preclinical data, these findings indicate that the genetic modification of *S. salivarius* K12 did not compromise its safety profile, supporting its continued nutritional application and future clinical development.

## 5 Conclusion

This exploratory randomized, double-blind, placebo-controlled trial demonstrates that the genetically modified *S. salivarius* eK12 strain, administered orally at 1 × 10^10^ CFU/day for 28 days—at a dosage deliberately selected to be approximately tenfold higher than the declared daily dose of *S. salivarius* K12 as a conservative high-dose safety challenge—is safe and well-tolerated in healthy adults. No adverse clinical, laboratory, microbiological, or inflammatory effects were observed during the 4-month monitoring period. Complementary GLP preclinical studies, including the Ames test (genotoxicity), acute oral toxicity (OECD 423), and a 90-day repeated-dose oral toxicity study (OECD 408), also revealed no adverse findings. Collectively, these results indicate that the genetically modified *S. salivarius* eK12 strain is clinically safe and well-tolerated in humans under the tested conditions, supporting its safe nutritional use and further clinical development.

## Data Availability

The original contributions presented in the study are included in the article/Supplementary material, further inquiries can be directed to the corresponding authors.
