# Supplementary material for: Study to evaluate the safety and tolerability of Streptococcus salivarius eK12, a genetically modified strain derived from the oral probiotic S. salivarius K12: Results from a randomized, double-blind, placebo-controlled, parallel-group clinical trial
Source: Front Nutr. 2025 Dec 8;12:1701611. doi: 10.3389/fnut.2025.1701611 (PMC12719284; doi:10.3389/fnut.2025.1701611)
Supplement: Supplementary file 1 [file Data_Sheet_1.zip › Supplementary Information.pdf]

Supplementary Information

Supplementary Table 1

Comparison of complete blood count (CBC) parameters between eK12 (n = 13) and Placebo (n = 16) groups at baseline, 1-month, and 4-month follow-up.

Parameter	Ref. Range	eK12, Baseline	eK12, 1-month	eK12, 4-month	Placebo, Baseline	Placebo, 1-month	Placebo, 4-month
Hb (g/dL)	12 – 16	13.6 ± 1.9	13.2 ± 2.0	13.6 ± 2.3	13 ± 1.8	13 ± 1.5	13 ± 2
HCT (%)	36 – 50	40.3 ± 5.3	39.3 ± 5.2	40.0 ± 6.0	40.6 ± 4.9	37.8 ± 4.5	39.1 ± 5.5
RBC ($\times 10^6$/μL)	4.3 – 5.9	5.13 ± 0.50	4.95 ± 0.60	4.92 ± 0.70	5.22 ± 0.49	4.86 ± 0.68	4.89 ± 0.74
MCV (fL)	78 – 98	79.8 ± 7.3	79.2 ± 8.5	80.3 ± 8.6	80.6 ± 7.2	80.1 ± 8.3	81.0 ± 8.2
MCH (pg)	27 – 32	26.9 ± 2.4	27.5 ± 2.6	27.6 ± 3.1	26.8 ± 2.7	27.1 ± 2.8	27.5 ± 2.9
MCHC (g/dL)	32 – 36	33.1 ± 1.1	33.2 ± 1.2	33.4 ± 1.3	33.0 ± 1.3	33.2 ± 1.4	33.3 ± 1.2
WBC ($\times 10^3$/μL)	4 – 10	6.7 ± 1.8	7 ± 2.1	8 ± 2.5	6.9 ± 1.4	7.5 ± 2.0	7.7 ± 1.8
Neutrophils (%)	40 – 75	52.3 ± 11.1	53.7 ± 10.8	55.0 ± 12.2	51.6 ± 7	55.8 ± 7	56.2 ± 10.0
Lymphocytes (%)	20 – 45	35.1 ± 10.6	34.6 ± 8.7	34.0 ± 10	36.3 ± 6	34.1 ± 6	33.4 ± 8
Monocytes (%)	2 – 10	7.6 ± 1.2	8 ± 1.3	7.5 ± 2	8 ± 2	7.4 ± 1.7	6.7 ± 1.7
Eosinophils (%)	2 – 6	4 ± 2	4 ± 1	3.5 ± 2	3.5 ± 1.5	2.8 ± 1	2.8 ± 1
Basophils (%)	< 1	0.7 ± 0.2	0.6 ± 0.2	0.7 ± 0.4	0.6 ± 0.4	0.5 ± 0.2	0.7 ± 0.5
Platelet Count ($\times 10^3$/μL)	150 – 400	328 ± 72	245 ± 95	251 ± 66	316 ± 95	254 ± 86	243 ± 85

Values are presented as mean ± SD. Hb = Hemoglobin; HCT = Hematocrit; RBC = Red blood cells; MCV = Mean corpuscular volume;

MCH = Mean corpuscular hemoglobin; MCHC = Mean corpuscular hemoglobin concentration; WBC = White blood cells .

Supplementary Table 2

Liver function test (LFT) parameters in eK12 (n = 13) and Placebo (n = 16) groups at baseline, 1-month, and 4-month follow-up.

Parameter (ref. range)	Ref. Range	eK12, Baseline	eK12, 1-month	eK12, 4-month	Placebo, Baseline	Placebo, 1-month	Placebo, 4-month
Serum Bilirubin (Total) mg/dL	0.1 - 1	0.42 ± 0.22	0.30 ± 0.16	0.47 ± 0.41	0.37 ± 0.33	0.50 ± 0.52	0.48 ± 0.36
Serum Bilirubin (Direct) mg/dL	<= 0.30	0.12 ± 0.09	0.12 ± 0.06	0.15 ± 0.08	0.11 ± 0.07	0.16 ± 0.13	0.15 ± 0.12
Serum Bilirubin (Indirect) mg/dL	0.25 - 0.90	0.30 ± 0.18	0.19 ± 0.11	0.32 ± 0.35	0.26 ± 0.26	0.34 ± 0.39	0.33 ± 0.25
Serum ALT (SGPT) U/L	<45	19.0 ± 9.4	21.5 ± 11.9	32.6 ± 19.0	20.7 ± 8.1	22.0 ± 14.0	36.5 ± 18.6
Serum ALP U/L	42 - 129	98.1 ± 31.2	111.6 ± 34.1	118.5 ± 44.1	82.8 ± 20.7	82.0 ± 26.3	90.4 ± 21.7
Serum GGT U/L	8-61	14.6 ± 7.6	20.7 ± 19.1	21.0 ± 17.8	17.25 ± 11.0	20.8 ± 11.0	20.0 ± 12.0
Serum AST (SGOT) U/L	<40	21.38 ± 4.72	24.08 ± 11.23	29.08 ± 11.06	25.06 ± 7.13	25.19 ± 8.95	33.62 ± 12.44

Values are presented as mean ± SD. ALT = alanine aminotransferase (SGPT); ALP = alkaline phosphatase; GGT = gamma-glutamyl transferase; AST = aspartate aminotransferase (SGOT).

Supplementary Table 3

Renal function tests (RFTs) parameters in eK12 (n = 13) and Placebo (n = 16) groups at baseline, 1-month, and 4-month follow-up.

Parameter	Ref. range	eK12 Baseline	eK12, 1-month	eK12, 4-month	Placebo, Baseline	Placebo, 1-month	Placebo, 4-month
Serum Creatinine mg/dL	0.70–1.20	0.76 ± 0.21	0.74 ± 0.22	0.81 ± 0.17	0.72 ± 0.14	0.74 ± 0.13	0.80 ± 0.21
Blood Urea mg/dL	15–50	21.23 ± 9.21	22.15 ± 6.62	24.46 ± 8.05	20.81 ± 5.00	22.12 ± 5.70	21.62 ± 6.44

Values are shown as mean ± SD.

Supplementary Table 4

Comparison of Serum Electrolytes between eK12 (n = 13) and Placebo (n = 16) groups at baseline, 1-month, and 4-month follow-up.

Parameter	Ref. Range	eK12 Baseline	eK12, 1-month	eK12, 4-month	Placebo Baseline	Placebo, 1month	Placebo, 4-month
Sodium (mEq/L)	136 – 145	139.8 ± 2.7	139.6 ± 2.0	139.1 ± 2.5	140.6 ± 2.0	140.1 ± 2.5	139.6 ± 2.4
Potassium (mEq/L)	3.5 – 5.5	4.5 ± 0.5	4.1 ± 0.4	4.2 ± 0.4	4.4 ± 0.3	4.2 ± 0.4	4.3 ± 0.5
Chloride (mEq/L)	98 – 107	103.4 ± 3.0	101.7 ± 2.4	102.5 ± 2.8	103.9 ± 2.8	101.6 ± 2.0	102.3 ± 2.5
Bicarbonate (mEq/L)	25 – 29	24.7 ± 3.3	25.0 ± 2.5	25.0 ± 2.9	24.5 ± 2.9	25.5 ± 2.5	25.1 ± 2.8

Values are presented as mean ± SD.

Supplementary Table 5

Comparison of HbA1c (%) between eK12 (n = 13) and Placebo (n = 16) groups at baseline, 1-month, and 4-month follow-up.

Parameter	Ref. range	eK12 Baseline	eK12, 1-month	eK12, 4-month	Placebo, Baseline	Placebo, 1-month	Placebo, 4-month
HbA1c %	4.8-5.6	5.4 ± 0.5	5.4 ± 0.5	5.2 ± 0.3	5.3 ± 0.3	5.3 ± 0.5	5.4 ± 0.8

Values are presented as mean ± SD. HbA1c = Glycated hemoglobin

Supplementary Table 6

Thyroid function parameters in eK12 (n = 13) and Placebo (n = 16) groups at baseline, 1-month, and 4-month follow-up.

Parameter	Ref. Range	eK12 Baseline	eK12, 1-month	eK12, 4-month	Placebo Baseline	Placebo, 1month	Placebo, 4-month
T3 ng/ml	0.8 - 2.0	1.0 ± 1.1	1.0 ± 0.3	0.9 ± 0.1	1.0 ± 0.1	1.0 ± 0.1	0.9 ± 0.2
T4 µg / dl	5.1-14.1	7.7 ± 1.1	7.3 ± 1.1	7.6 ± 1.5	7.1 ± 1.1	6.8 ± 1.3	6.9 ± 1.3
TSH µIU/mL	0.27-4.2	1.5 ± 0.8	1.7 ± 0.6	1.5 ± 0.6	1.9 ± 1.2	1.8 ± 1.2	1.6 ± 0.6

Values are presented as mean ± SD. T3 = Triiodothyronine; T4 = Thyroxine; TSH = Thyroid-stimulating hormone.

Supplementary Table 7

Urinalysis parameters in eK12 (n = 13) and Placebo (n = 16) groups at baseline, 1-month, and 4-month follow-up

Parameter	Ref. Range	eK12 Baseline	eK12, 1-month	eK12, 4-month	Placebo Baseline	Placebo, 1-month	Placebo, 4-month
Specific Gravity	1.005 – 1.030	1.03 ± 0.01	1.02 ± 0.01	1.02 ± 0.01	1.02 ± 0.01	1.0 ± 0.1	1.02 ± 0.01
pH	5.0 – 8.0	6.0 ± 0.4	6.0 ± 0.5	6.0 ± 0.7	6.2 ± 0.6	6.2 ± 0.6	6.0 ± 0.7

Values are presented as mean ± SD.

Supplementary Table 8

Serum inflammatory markers levels in eK12 (n = 13) and Placebo (n = 16) groups at baseline, 1-month, and 4-month follow-up.

Parameter	Ref. range	eK12 Baseline	eK12, 1-month	eK12, 4-month	Placebo, Baseline	Placebo, 1-month	Placebo, 4-month
CRP mg/dL	< 0.5	0.24 ± 0.23	0.20 ± 0.14	0.41 ± 0.62	0.41 ± 0.98	0.19 ± 0.27	0.015 ± 0.17
PCT ng/ml	<0.07	0.03 ± 0.02	0.04 ± 0.03	0.04 ± 0.03	0.03 ± 0.01	0.02 ± 0.01	0.02 ± 0.01
Ferritin mg/dL	30 – 400	97.2 ± 82.8	93.3 ± 88.3	121.6 ± 116.4	72.9 ± 56.4	66.8 ± 52.5	92.8 ± 64.8

Values are presented as mean ± SD. CRP = C-reactive protein; PCT = procalcitonin.

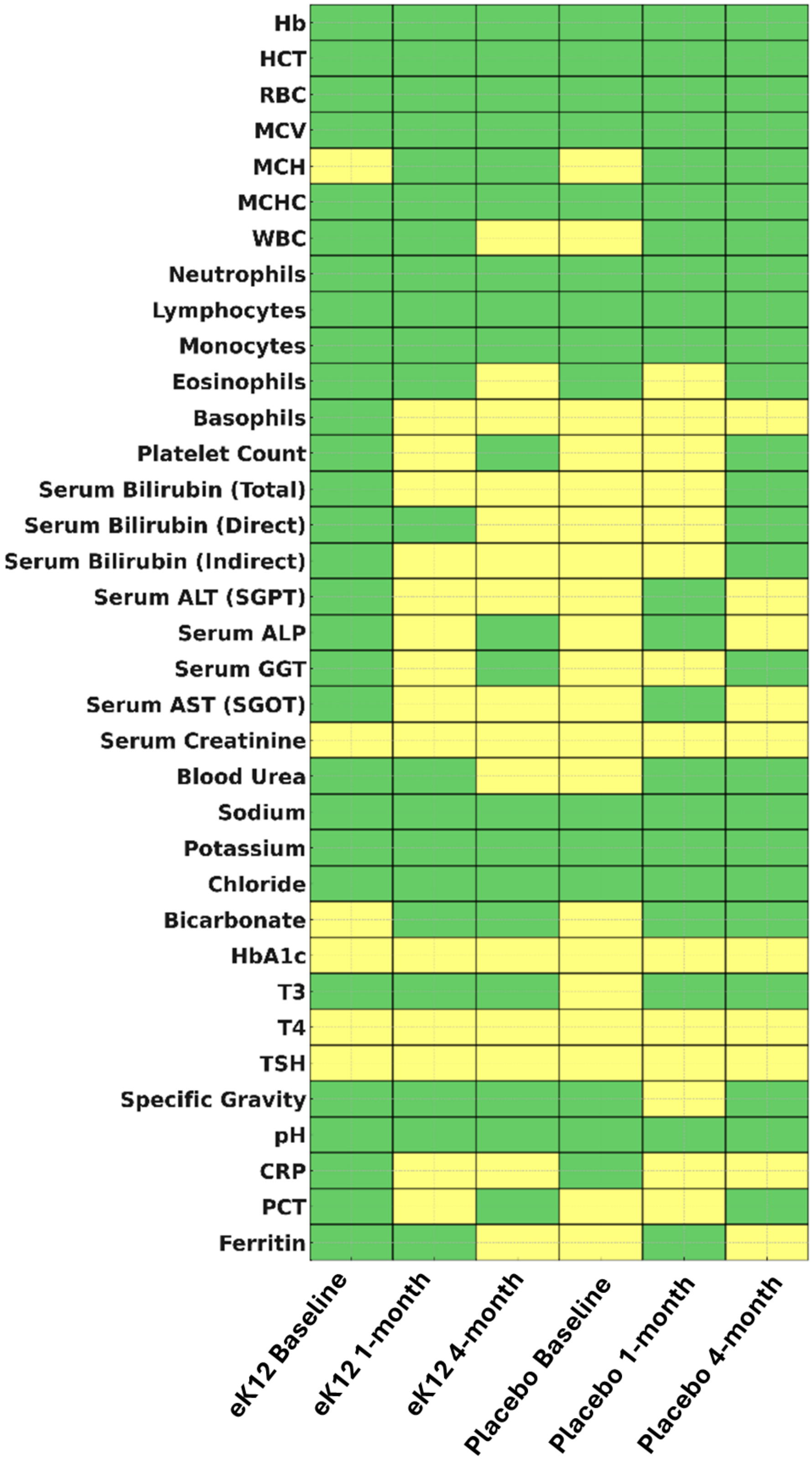

Supplementary Figure 1. Cluster heatmap of hematological, biochemical, and urinary parameters in healthy adults receiving *Streptococcus salivarius* eK12 or placebo. Each cell represents the group mean value at baseline, 1-month, or 4-month follow-up. Green indicates values within the corresponding normal reference range; yellow denotes

minor, non-pathological variations without clinical significance. No parameter showed deviations outside reference limits, confirming the overall safety of *S. salivarius* eK12 administration. Abbreviations: Hb – Hemoglobin; HCT – Hematocrit; RBC – Red Blood Cell Count; MCV – Mean Corpuscular Volume; MCH – Mean Corpuscular Hemoglobin; MCHC – Mean Corpuscular Hemoglobin Concentration; WBC – White Blood Cell Count; ALT – Alanine Aminotransferase; AST – Aspartate Aminotransferase; ALP – Alkaline Phosphatase; GGT – Gamma-Glutamyl Transferase; AST –Aspartate Aminotransferase; HbA1c – Glycated Hemoglobin; T3 – Triiodothyronine; T4 – Thyroxine; TSH – Thyroid-Stimulating Hormone; pH – Urine Acidity/Alkalinity; CRP – C-Reactive Protein; PCT – Procalcitonin.
